# Covalent Immobilization of Pectinase on Functionalized Magnetic Nanoparticles for Improved Catalytic Stability and Its Application in Grape Juice Clarification

**DOI:** 10.1002/fsn3.72110

**Published:** 2026-07-15

**Authors:** Olgun Cirak, Mesut Işık, Alev Akpinar Borazan, Şükrü Beydemir

**Affiliations:** ^1^ Department of Biotechnology, Graduate Education Institute Bilecik Şeyh Edebali University Bilecik Turkey; ^2^ Department of Hospitality Restaurant and Catering Services, Vocational School Bilecik Şeyh Edebali University Bilecik Turkey; ^3^ Department of Bioengineering, Faculty of Engineering Bilecik Şeyh Edebali University Bilecik Turkey; ^4^ Department of Chemistry Engineering, Faculty of Engineering Bilecik Şeyh Edebali University Bilecik Turkey; ^5^ Department of Biochemistry, Faculty of Pharmacy Anadolu University Eskişehir Turkey

**Keywords:** fruit juice clarification, immobilization, magnetic nanoparticle (MNP), pectinase

## Abstract

In this study, the commercial pectinase enzyme derived from *Aspergillus niger* which is of critical importance in the food industry was covalently immobilized onto surface‐functionalized magnetic nanoparticles to maximize its operational stability and reusability. The magnetic nanoparticles (MNPs) used as the carrier material were synthesized via the co‐precipitation method. To enhance their chemical and physical stability, they were coated with silica using tetraethyl orthosilicate (TEOS), and amino groups were subsequently incorporated into their surfaces using 3‐aminopropyl triethoxysilane (APTES). Pectinase was covalently immobilized onto these modified nanoparticles via glutaraldehyde, a cross‐linking agent, and the successful synthesis of the nanobiocatalyst was confirmed by FT‐IR and SEM–EDX analyses. As a result of catalytic performance evaluations, it was determined that the immobilization process did not cause any shift in the enzyme's optimal temperature (50°C) or optimal pH (pH 5.0). In contrast, the developed magnetic nanobiocatalyst exhibited a significantly superior profile compared to the free enzyme in terms of thermal stability, pH tolerance, and long‐term storage stability. Operational stability (reusability) tests revealed that the immobilized pectinase could be easily recovered using an external magnetic field due to strong covalent bonds and managed to retain 81% of its initial activity even after 20 consecutive reaction cycles. The industrial applicability of the synthesized nanobiocatalyst was tested in a grape juice clarification process, resulting in a dramatic 82% reduction in the turbidity of the fruit juice. In conclusion, given its high catalytic performance and exceptional operational stability, this developed magnetic enzyme system has been shown to offer a sustainable biocatalytic solution with high potential for the fruit juice and food processing industries.

## Graphical abstract

Covalently immobilized *Aspergillus niger* pectinase on silica‐ and amino‐functionalized magnetic nanoparticles exhibited enhanced thermal, pH, storage, and operational stability without altering its optimum reaction conditions. The magnetic nanobiocatalyst was easily recovered using an external magnetic field, retained high activity after repeated use, and efficiently clarified grape juice, demonstrating its potential as a sustainable and reusable biocatalyst for fruit juice and food processing applications.

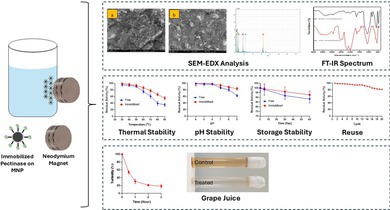

## 1 Introduction

Enzymes are environmentally friendly, protein‐based biocatalysts that play a role in cellular metabolism and function with high catalytic efficiency, specificity, and selectivity under moderate reaction conditions (physiological pH, temperature, and atmospheric pressure) (Kim et al. 2008). Thanks to these superior properties, they are increasingly being preferred as alternatives to chemical catalysts in many industrial sectors today, such as food, chemicals, pharmaceuticals, paper, and textiles (Samiur Rashid et al. 2021). However, the direct use of free (soluble) enzymes in large‐scale industrial processes creates serious technological and economic bottlenecks, such as low operational stability, short shelf life, sensitivity to extreme pH and temperature fluctuations, and the inability to separate them from the product after the reaction. The inability to recover these high‐cost biocatalysts from the reaction medium and their single‐use nature increases process costs, thereby limiting their industrial feasibility (Maghraby et al. 2023).

Enzyme immobilization technologies have been developed to overcome these limitations and maximize the potential of enzymes in industrial applications (Homaei et al. 2013; Robescu and Bavaro 2025). Immobilization is based on the principle of trapping or binding the enzyme to an insoluble support material (carrier) in the reaction medium through physical or chemical means. Compared to their free forms, immobilized enzymes offer higher structural stability, expanded environmental tolerance, long‐term storage stability, and most importantly the ability to be easily recovered from the reaction medium and reused multiple times (sequentially), thereby preventing product contamination (Homaei et al. 2013). Although various techniques such as adsorption, entrapment, and cross‐linking are described in the literature, the covalent binding method stands out because it forms permanent chemical bonds between the enzyme and the carrier matrix (Mosafa et al. 2014). This method imparts physical rigidity to the enzyme's three‐dimensional conformation through a multi‐point covalent bonding mechanism. This prevents protein denaturation and prevents the enzyme from leaching from the support during reaction or washing steps (Bilal, Asgher, et al. 2018).

The success of covalent immobilization depends largely on the mechanical, physicochemical, and morphological properties of the selected support material (Qi et al. 2020). In recent years, with advances in nanotechnology and materials science, nanostructured materials with high specific surface area and low mass transfer resistance (diffusion limitation) have attracted attention as enzyme carriers (Oke et al. 2023). Among these nanomaterials, iron oxide‐based superparamagnetic nanoparticles (Fe_3_O_4_) hold a unique position in biocatalyst design. Magnetic nanoparticles (MNPs), in addition to their superior biocompatibility, low toxicity, biodegradability, and high enzyme loading capacity, have the ability to be effortlessly, cost‐effectively, and without loss separated from the reaction medium within seconds using an external magnetic field (magnet), thereby providing significant flexibility for industrial operations (Mosafa et al. 2014).

However, uncoated magnetic nanoparticles tend to agglomerate in solution due to their high surface energies and magnetic dipole–dipole interactions, and when exposed to oxygen in the air, they may oxidize and lose their structural and magnetic properties. Surface modification of MNP is a critical step to overcome these structural limitations, prevent agglomeration, and create reactive sites (functional groups) on the surface suitable for covalent enzyme binding (Bilal, Zhao, et al. 2018). Typically, coating the magnetic core with a protective silica (SiO_2_) shell using tetraethyl orthosilicate (TEOS) (core‐shell structure) provides the particles with chemical stability and an abundance of hydrophilic silanol (‐OH) groups on the surface. Next, primary amine (‐NH_2_) groups are incorporated into the silica surface using a silanization agent such as 3‐aminopropyl triethoxysilane (APTES) (Aslani et al. 2018; Samiur Rashid et al. 2021). This functionalized surface is activated by a bifunctional cross‐linking agent called glutaraldehyde (GA). The GA molecule ensures excellent immobilization through covalent Schiff base (‐C=N‐) interactions by reacting one of its two aldehyde groups with the amine groups on the support surface and the other with the free amino groups found in the lysine residues on the enzyme surface (Başaran and Işık 2025; Işık 2020).

One of the enzymes that stands to benefit the most from these advanced immobilization platforms in food biotechnology is pectinase. Pectinase (E.C. 3.2.1.15) is an enzyme widely used in industry that hydrolyzes pectin of the primary polysaccharide components of the plant cell wall into galacturonic acid by cleaving the α‐1,4‐glycosidic bonds in its structure (Li et al. 2007). In particular, during the production of fresh‐pressed fruit juices, pectin (at a concentration of 0.9%–1.5%) is released into the medium as a result of cell lysis and forms a colloidal dispersion, causing the fruit juice to become turbid (Tapre and Jain 2014). Pectinase is used as a standard practice in the fruit juice industry to eliminate this undesirable cloudiness, reduce fruit juice viscosity, and increase extraction and filtration efficiency (Amin et al. 2023; Sojitra et al. 2016, 2017). In traditional production lines, free enzymes are subjected to harsh processes, such as thermal inactivation, to stop the reaction or remove the enzyme from the product. This compromises the natural nutritional value (vitamins) and delicate aroma profile of fruit juices (Kuddus 2019). Therefore, the development of a magnetic pectinase nanobiocatalyst that can be used directly and safely in fruit juice production—one that is resistant to thermal conditions and can be removed from the system using a magnet for repeated use is of great importance to the industry.

Designed in light of these scientific and technological requirements, the aim of this study is to successfully immobilize a commercially available pectinase enzyme derived from *Aspergillus niger* onto surface‐functionalized iron oxide magnetic particles using a covalent bonding strategy, in order to maximize its operational stability and reusability. The immobilization was verified using Fourier‐Transform Infrared Spectroscopy (FT‐IR) and Scanning Electron Microscopy (SEM–EDX) techniques. Within the scope of this article, the kinetic behavior (*K*_m_ and *V*_max_), temperature profiles, pH stability, and storage (shelf life) stability of pectinase in both free and immobilized forms were comprehensively investigated and compared. The industrial practicality of this robust, high‐performance nanobiocatalyst system was demonstrated by its operational stability (reusability) across successive cycles and its high turbidity removal performance in grape juice clarification applications.

## 2 Materials and Methods

### 2.1 Materials

Pectinase from *Aspergillus niger* (3.2.1.15), apple pectin (galacturonic acid min. 58%) and α‐D‐galacturonic acid was procured from TCI company. Dinitrosalicylic acid (DNS), ammonium solution (NH_3_, 25%), sodium potassium tartrate, glutaraldehyde solution (25%) (GA), tetra‐ethyl orthosilicate (TEOS), 3‐amino propyl tri‐ethoxy silane (APTES), coomassie brilliant blue G250, bovine serum albumin (BSA), ferric chloride hexahydrate (FeCl_3_·6H_2_O) and ferrous chloride tetrahydrate (FeCl_2_·4H_2_O) and sodium acetate were purchased from Merck Chemicals Co. All other chemicals were of analytical grade and procured from reliable sources.

### 2.2 Synthesis of Magnetic Nanoparticles (MNPs)

MNPs were synthesized by making some modifications to the traditional co‐precipitation method described in the literature (Başaran and Işık 2025; Mariño et al. 2023; Rusu et al. 2022). FeCl_3_·6H_2_O and FeCl_2_·4H_2_O were dissolved in deionized water in a 2:1 M ratio. The solution was heated to 80°C while being stirred continuously. NH_4_OH was added to the mixture to induce the formation of a black magnetite (Fe_3_O_4_) precipitate. The resulting black precipitate was collected using a magnet, and the supernatant was removed from the mixture using a pipette. Finally, the magnetite precipitate was dried in an oven at 50°C.

### 2.3 Functionalization of MNPs

The method was carried out with some modifications to the previously described approach for functionalizing MNP surfaces (Başaran and Işık 2025; Mariño et al. 2023; Rusu et al. 2022). 1 g of MNP was mixed with 150 mL of ethanol and 30 mL of distilled water, then sonicated for 30 min. Next, 20 mL of NH_4_OH was slowly added to the mixture, which was then stirred on a magnetic stirrer for 30 min. After adding 5 mL of TEOS dropwise, the mixture was stirred on a magnetic stirrer for 12 h. The TEOS‐coated MNPs were then collected using a strong neodymium magnet, and the supernatant was removed using a pipette. The MNPs were then washed three times with distilled water and ethanol, respectively. The washed MNPs were dispersed by sonicating them in 200 mL of ethanol for 30 min. Next, 0.5 mL of NH_4_OH was added to the mixture in the magnetic stirrer, followed by the dropwise addition of 2 mL of APTES. The mixture was then stirred continuously on the magnetic stirrer for 12 h. After removing the supernatant, the particles were washed with distilled water and ethanol.

The surface‐functionalized MNPs were dispersed in 100 mL of 0.1 M phosphate buffer (pH 7.0) using an ultrasonic bath. Next, 15 mL of glutaraldehyde was added, and the mixture was stirred at room temperature for 3 h on a magnetic stirrer. The sample was then washed three times with phosphate buffer to remove unreacted glutaraldehyde.

### 2.4 Pectinase Immobilization

200 mg of MNP activated with glutaraldehyde and 20 mg of pectinase powder were incubated overnight at +4°C in 50 mL of phosphate buffer (pH 6) (Nouri and Khodaiyan 2020; Sojitra et al. 2017). Subsequently, the magnetic nanoparticles were separated from the reaction mixture using a magnet and washed with buffer. The immobilization yield was determined by measuring the difference between the initial protein amount and the protein amounts remaining in the supernatant and the washing buffer. Protein concentration was determined using the Bradford method with standard bovine serum albumin (Bradford 1976).

### 2.5 Pectinase Activity Assay

Both free and immobilized pectinase activity were determined using the DNS method measured spectrophotometrically (Miller 1959). This method is based on quantitatively measuring the color development following the DNS reaction and subtracting the sugars released by enzyme activity. For free pectinase, the enzyme was incubated with apple pectin (0.5%, w/v) for 20 min, and the reaction was terminated by adding 500 μL of DNS reagent. The mixture was heated in a water bath for 10 min and then cooled to room temperature. Absorbance was measured spectrophotometrically at 540 nm. 5 mg of dry immobilized MNP was used for immobilized enzyme activity measurements. 1 Unit (U) of pectinase activity is the amount of enzyme that releases the equivalent of 1 μmol of D‐galacturonic acid per minute under optimal conditions (pH 5 and 50°C). A calibration curve was obtained using D‐galacturonic acid in the concentration range of 0.0–0.5 mg/mL.

### 2.6 Carrier Characterization

Fourier transform infrared (FT‐IR) spectroscopy was obtained using a Perkin Elmer/Spectrum 100 spectrometer covering wavenumbers between 400 and 4000 cm^−1^. Morphological and elemental analysis was performed using a ZEISS/Supra 40 VP scanning electron microscope (SEM–EDX) equipped with an EDX instrument.

### 2.7 Optimum Temperature Assay

The optimum temperature for pectinase activity was determined by measuring the activity of free pectinase and immobilized pectinase in the range of 30°C–90°C at pH 5 (acetate buffer). For better comparison, the highest activity value was set as 100% (optimum temperature). Activity values at all other temperatures were normalized relative to the highest value and multiplied by 100 to convert them to relative activity percentages. Results correspond to the mean ± SD of triplicates.

### 2.8 Optimum pH Assay

The optimal pH of the pectinase enzyme was determined by measuring free and bound pectinase activity at 50°C using an acetate buffer (pH 4–5), a phosphate buffer (pH 6–7), or a Tris buffer (pH 8–9). For better comparison, enzyme activity at the highest (optimum pH) level was set as 100%, and relative activity (the ratio of the observed value to the optimum value) was used for comparison. Results correspond to the mean ± SD of triplicates.

### 2.9 Kinetic Parameters

As described above, the kinetic parameters of both free and immobilized enzymes were determined using various pectin solution concentrations (0.25–5.0 mg/mL) in the experimental mixtures under optimum experimental conditions (pH 5 and 50°C). To eliminate the effects of potential enzyme inactivation and steric hindrance following immobilization, the kinetic parameters (*K*_m_ and *V*_max_) were determined by normalizing the initial activities of the free and immobilized pectinase. The free enzyme was diluted so that it exhibited the same initial reaction rate as 5 mg of immobilized pectinase under optimal experimental conditions. The *K*_m_ and *V*_max_ values of free and immobilized pectinase were calculated from the nonlinear regression fitting of the initial reaction rates corresponding to different pectin concentrations using the Graph Pad Prism (Version 8.0.2) program.

### 2.10 Thermal Stability Assay

Free and immobilized enzymes were incubated in pH 5 buffers at different temperatures (30°C–90°C) for 3 h without the addition of substrate. After cooling to room temperature, pectin was added, and enzyme activity was measured under optimal conditions. The initial activity of the enzymes not subjected to heat treatment was taken as 100%. Subsequently, the residual activity (%) for each temperature point was calculated as the ratio of the activity after 3 h to the initial activity. Results correspond to the mean ± SD of triplicates.

### 2.11 pH Stability Study

Free and immobilized enzymes were incubated for 3 h (at 50°C) at different pH values (pH 4–9) without the addition of substrate. Pectin was then added, and enzyme activity was measured under optimal conditions (pH 5 and 50°C). The initial activity measured under optimum conditions was taken as 100%. The residual activity (%) for each pH point was then calculated as the ratio of the activity after 3 h to the initial activity. Results correspond to the mean ± SD of triplicates.

### 2.12 Storage Stability Assay

The storage times of both free and immobilized pectinase in substrate‐free buffer solutions were evaluated by measuring activity at the end of days 0, 5, 30, and 60 at 4°C, respectively. Subsequently, the residual activity was calculated as a percentage of the initial activity (day 0). Results correspond to the mean ± SD of triplicates.

### 2.13 Reusability Assay of the Immobilized Pectinase

The reusability of immobilized pectinase was investigated in hydrolysis cycles conducted at pH 5 and 50°C using apple pectin (0.5%, w/v) as the substrate. While the supernatant was used to determine the galacturonic acid concentration via a DNS assay, the immobilized pectinase was resuspended in fresh buffer solution, and the reaction was initiated by adding pectin. The initial activity of the immobilized enzyme was assumed to be 100%. The enzyme activity measured after each subsequent usage cycle was calculated as a percentage of the initial activity.

### 2.14 Clarification of Grape Juice

The performance of immobilized pectinase in clarifying red grape juice was investigated. Fresh grapes purchased from the local market were washed with tap water and then rinsed with distilled water. The grapes were then placed in a muslin cloth and squeezed to extract the grape juice. The fresh grape juice was centrifuged at 4500 rpm for 20 min. A portion of the resulting supernatant was set aside as a control and subjected to the same conditions as the grape juice treated with the immobilized enzyme. The remaining portion of the grape juice (50 mL) was mixed with immobilized pectinase (20 mg) and treated at 50°C for 3 h. After the enzymatic treatment, the clarity of the juice was determined spectrophotometrically (at 660 nm) by measuring turbidity (Dal Magro et al. 2016). Results correspond to the mean ± SD of triplicates.

## 3 Results and Discussion

### 3.1 Immobilization Yield

One of the most fundamental parameters determining the industrial potential, cost‐effectiveness, and catalytic capacity of the developed magnetic nanobiocatalyst is the immobilization yield (Lu et al. 2022). In this study, the amount of pectinase bound to functionalized magnetic nanoparticles (MNP) was quantitatively determined by calculating, using the Bradford method, the difference between the initial protein concentration added to the reaction medium and the amounts of free protein removed from the supernatant and washing buffers after the reaction (Bradford 1976; Kharazmi et al. 2020).

As a result of the analyses, the immobilization yield of the pectinase enzyme on the magnetic nanocarrier was determined to be 57%. This protein loading rate of 57% is consistent with the literature and is a highly satisfactory value, particularly for covalent immobilization procedures using bifunctional cross‐linking agents such as glutaraldehyde (Robescu and Bavaro 2025; Zucca and Sanjust 2014). Unlike methods such as physical adsorption or entrapment, in covalent binding strategies, only protein molecules capable of forming strong Schiff base interactions between the active aldehyde groups on the carrier surface and the lysine residues on the enzyme surface can be permanently retained on the surface (Sojitra et al. 2017; Trindade Ximenes et al. 2021). The calculated 57% yield represents not so much a numerical limitation as it does a genuine and permanent fraction of active enzyme that is multipoint attachment to the nanocarrier surface, carries no risk of leakage, and exhibits high conformational rigidity (Rodrigues et al. 2021).

### 3.2 Synthesis and Characterization of Support Material and Immobilization Process

**FIGURE 1:**
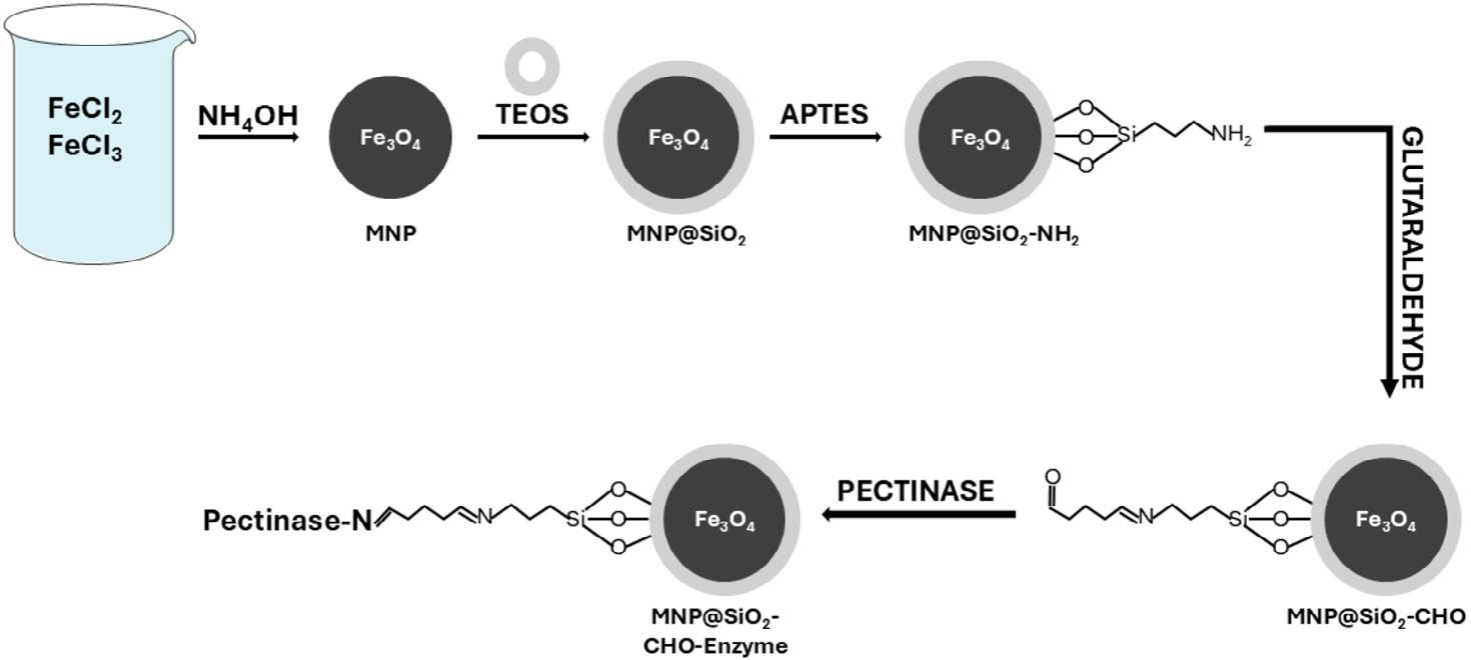
Synthesis of TEOS and APTES functionalized MNPs and pectinase immobilization.

The synthesis of a nanobiocatalyst designed to enhance the operational stability of the pectinase enzyme and ensure its reusability in industrial processes was carried out through a series of sequential modification steps (Figure 1). In the first stage, black magnetite (Fe_3_O_4_) nanoparticles exhibiting superparamagnetic properties were synthesized by co‐precipitation of iron(II) and iron(III) ions in a 1:2 M ratio in an alkaline medium (in the presence of ammonium hydroxide) (Behram et al. 2023). However, bare Fe_3_O_4_ nanoparticles tend to agglomerate easily due to their high surface energy and, when exposed to air, run the risk of oxidizing and losing their magnetic properties (Bilal, Zhao, et al. 2018). To overcome these limitations and impart chemical stability to the structure, the particles were coated with a silica (SiO_2_) shell with tetraethyl orthosilicate (TEOS). In addition to protecting the magnetic core, the silica coating process has imparted high hydrophilic properties to the particles by creating a large number of silanol (‐OH) groups on the surface and has provided strong anchorage points for the next modification step (Bilal, Zhao, et al. 2018). The surface of the silica‐coated magnetic nanoparticles was subjected to a silanization process using 3‐aminopropyltriethoxysilane (APTES) to allow covalent binding of the enzyme, and primary amine (−NH_2_) groups were incorporated into the particle surface (Muley et al. 2020). Glutaraldehyde, one of the most used homobifunctional cross‐linking agents for binding free enzymes to a support, was added to the reaction mixture at this stage. The glutaraldehyde molecule formed covalent Schiff base (‐C=N‐) bonds by reacting one of its two reactive aldehyde groups with the amine groups on the APTES‐functionalized particle surface and the other with the free primary amine groups on the lysine residues located on the outer surface of the pectinase molecule (Başaran and Işık 2025; Fang et al. 2016; Işık 2020).

The surface morphology and topographical characteristics of the synthesized functional magnetic nanocarriers and the final biocatalyst obtained after pectinase immobilization were examined using scanning electron microscopy (SEM).

**FIGURE 2:**
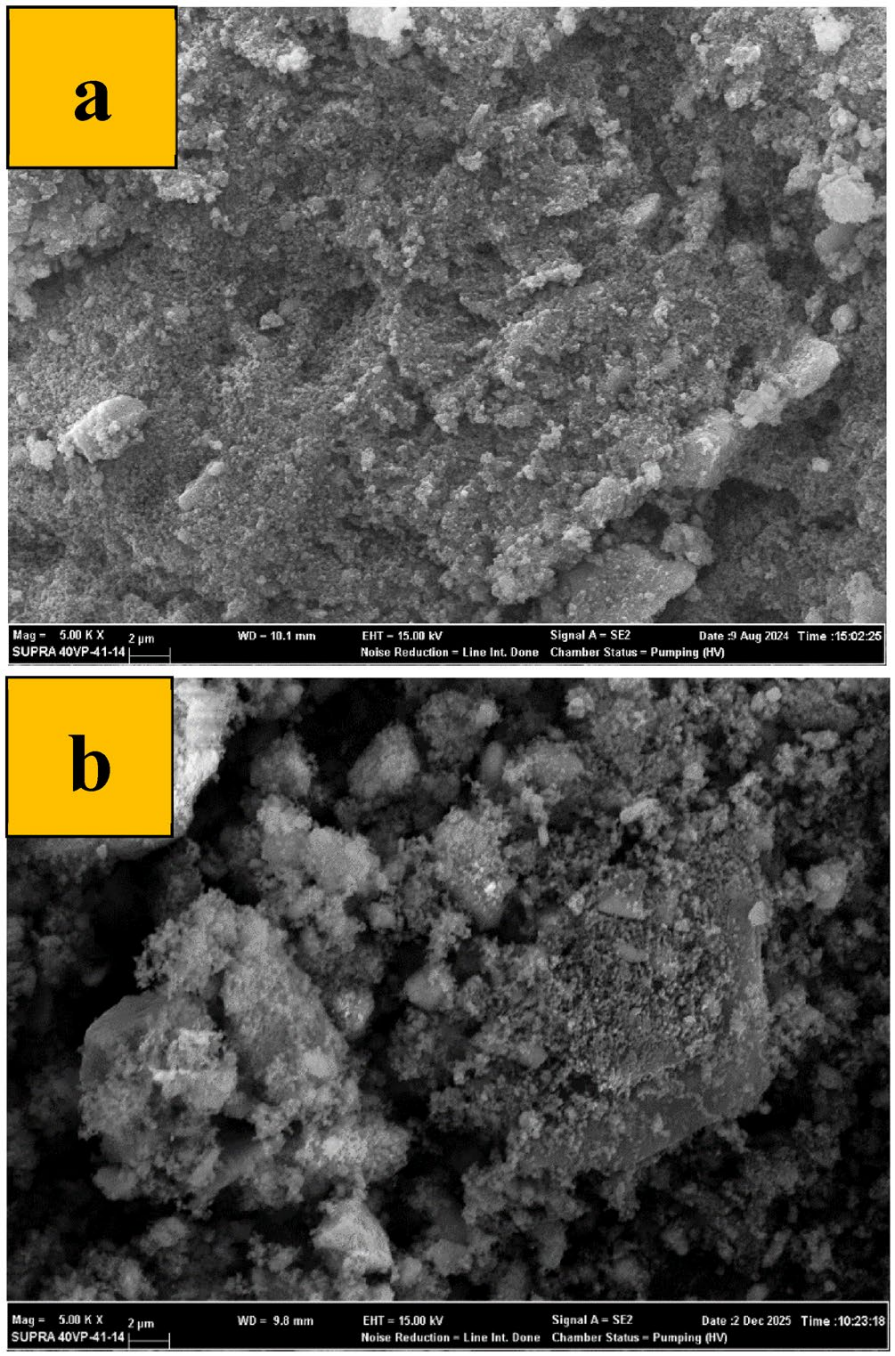
Comparative morphological analyses of immobilized pectinase obtained using scanning electron microscopy. (a) Surface topography of the nano‐carrier prior to immobilization. (b) Morphological changes in the nanobiocatalyst after the pectinase enzyme was immobilized.

Upon evaluation of the micrograph in Figure 2a, taken prior to immobilization, it is observed that the functionalized magnetic nanoparticles exhibit irregular structures with relatively distinct boundaries, clustered at the nanometric and submicrometric scales. Although the silica (SiO_2_) shell surrounding the iron oxide core and the subsequent APTES and glutaraldehyde (GA) modifications coated the particle surface, the granular and particulate character inherent to inorganic materials was largely preserved. This tendency of nanoparticles to agglomerate (cluster) with one another stems from the high specific surface energies at the nanoscale and the magnetic dipole–dipole interactions between the iron oxide cores.

The micrograph in Figure 2b, obtained after the pectinase enzyme was covalently immobilized onto the nanocarrier surface via the reactive free aldehyde groups of glutaraldehyde, reveals a quite distinct and characteristic morphological transformation. Following immobilization, the initially relatively distinct inorganic particle boundaries disappeared, giving way to a much rougher, denser, and amorphous organic macromolecular layer that filled the interparticle voids. This thick protein layer, formed by the covalent binding of enzyme molecules to the inorganic surface, has created a cloudy, sponge‐like, and highly agglomerated network structure that bridges the nanocarriers together.

This observed morphological change, along with the enhanced surface treatment and the presence of a dense organic coating enveloping the inorganic matrix, structurally confirms that pectinase molecules are not merely adsorbed onto the support material via weak physical bonds, but are strongly bound to the particle surface by covalent Schiff base (‐C=N ‐) interactions, as intended.

**FIGURE 3:**
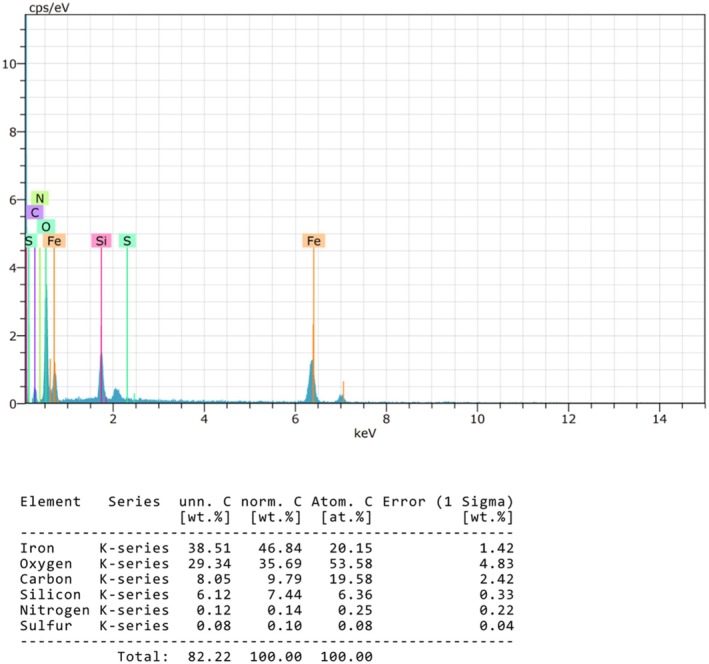
Energy dispersive X‐ray (EDX) spectrum and elemental profile of immobilized pectinase.

After immobilization with pectinase, the elemental composition of the magnetic nanobiocatalyst was examined using EDX spectroscopy (Figure 3). The analysis results clearly show the characteristic signals of the elements Fe, O, Si, C, N, and S. A quantitative evaluation based on normalized weight percentages revealed that iron had the highest mass percentage (46.84%), followed by oxygen (35.69%) and carbon (9.79%). A strong peak for Fe (approximately 6.4 keV) confirms the presence of a magnetic core; significant oxygen and carbon signals, meanwhile, support the formation of an organic layer and functional groups on the surface. Furthermore, the detection of 7.44% silicon indicates that the surface of the magnetic nanoparticles was successfully coated with a TEOS‐derived SiO_2_ layer. The nitrogen signal (0.14%), indicating successful APTES modification and subsequent enzyme binding, confirms the integration of ‐NH_2_ functional groups and protein molecules. On the surface, the presence of sulfur at a weight of 0.10% serves as a specific elemental marker supporting the preservation of the covalently bound enzymatic structure (cysteine/methionine residues) within the nanocarrier system.

Overall, the elemental profile obtained from the EDX spectrum chemically validates each stage of the functionalization and immobilization process. The signals from the C, N, and S elements specifically reflect the covalent bonding mechanism via the glutaraldehyde cross‐linking agent and the dense organic structure of the immobilized enzyme layer. The effective formation of the silica and metal oxide structure is confirmed by high mass percentages of oxygen and iron, while the varying ratios of carbon and nitrogen atoms clearly demonstrate the successful integration of organic biocatalytic components onto the surface. These findings demonstrate the successful architecture of the functionalized nanobiocatalyst.

**FIGURE 4:**
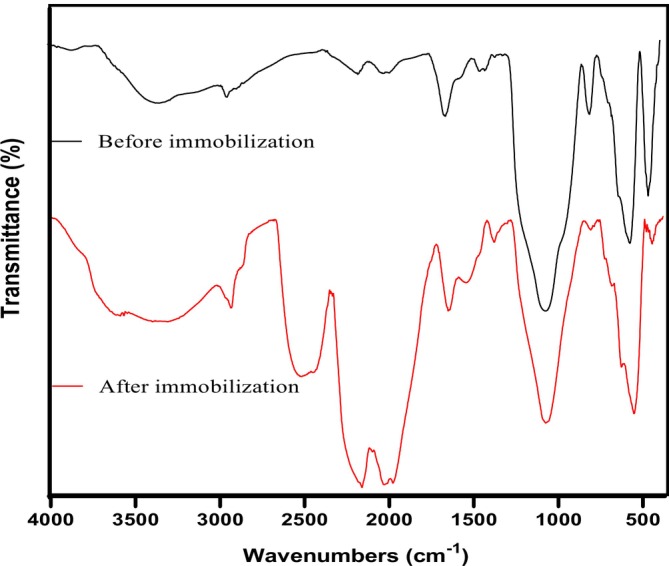
FT‐IR spectra of MNP@SiO_2_‐NH_2_‐GA before and after immobilization.

To evaluate the changes in the chemical structure of the functionalized magnetic nanoparticles, the FT‐IR spectra of the pre‐ and post‐immobilization samples were analyzed comparatively (Figure 4). FT‐IR spectra show distinct peaks reflecting functional groups within the materials. The pre‐immobilization spectrum (black line) contains characteristic bands indicating both silica coating and APTES‐derived amino functionalities. The prominent Si‐O‐Si asymmetric stretching band observed in the 1080–1100 cm^−1^ region and the Si‐O stretching mode around 790–800 cm^−1^ demonstrate the success of silica coating achieved with TEOS. The Fe‐O vibrational band observed around 560 cm^−1^ confirms the presence of a magnetic core. ‐NH_2_ groups, indicating APTES functionalization, are supported by N‐H bending vibrations in the 1550–1560 cm^−1^ region.

Following pectinase immobilization, the spectrum (red line) shows the emergence of new bands specific to protein structures and a significant change in the intensity of some bands. The band that becomes particularly prominent around 1630–1660 cm^−1^ is an amide I stretching vibration belonging to the enzyme protein structure and originates from C=O groups. The amide II band, appearing in the 1520–1550 cm^−1^ range, represents N‐H stretching modes associated with the immobilized enzyme layer. The addition of these two bands to the spectrum after immobilization strongly supports the successful binding of the enzyme to the particle surface.

On the other hand, the characteristic shift in the range of approximately 1630–1650 cm^−1^ is a typical chemical indicator pointing to glutaraldehyde‐mediated Schiff base (C=N) formation. Covalent bonding between aldehyde groups and ‐NH2 groups on the surface of APTES enhances the visibility of this C=N vibration, confirming the immobilization mechanism. Furthermore, the concentration of C‐H stretching bands around 2850–2950 cm^−1^ in the post‐immobilization spectrum can be attributed to increased organic layer formation on the surface. Overall, the FT‐IR results show that the magnetic core is silica‐coated, the surface acquires amine functionalities through APTES modification, glutaraldehyde activation occurs, and amide bonds in the enzyme structure are clearly visible after immobilization. These findings confirm that each stage of the immobilization process in the surface chemistry of the nanoparticles was successfully completed.

### 3.3 Optimum Temperature Assay

**FIGURE 5:**
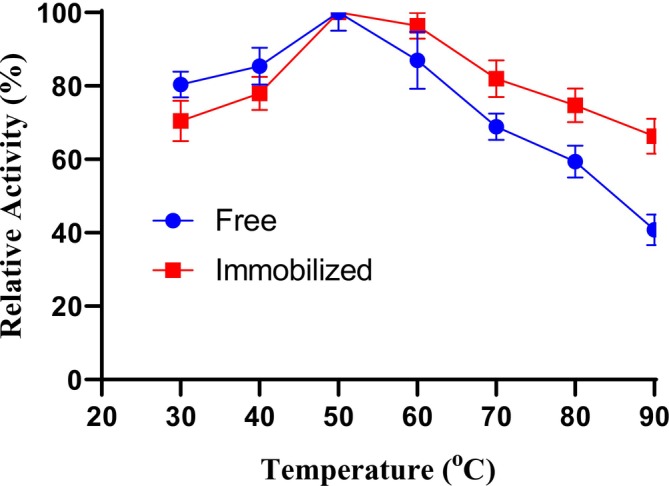
The effect of temperature on the relative activity of free and immobilized pectinase (data are presented as mean ± SD of triplicates).

The effect of reaction temperature on the catalytic performance of free and immobilized pectinase was investigated in the range of 30°C–90°C. As shown in Figure 5, based on the relative activity‐temperature profiles presented, it was determined that both enzyme forms reached maximum catalytic activity at 50°C and that the immobilization process did not cause a shift in the enzyme's optimal temperature (Behram et al. 2023; Kharazmi et al. 2020). However, upon examining the distribution of the obtained temperature profiles, it is clearly evident that the developed nanobiocatalyst exhibits a much broader catalytic tolerance range compared to the free enzyme, particularly at high temperatures. At high temperatures exceeding the optimum temperature, the immobilized enzyme retained a higher percentage of its activity compared to the free enzyme. For example, while the relative activity of the free enzyme dropped to approximately 87% at 60°C, the immobilized enzyme managed to retain 96% of its activity. As the temperature continued to rise, this difference became even more pronounced; while the activity of the free enzyme dropped to approximately 59% at 80°C and to 40% at an extreme temperature of 90°C, the immobilized pectinase continued to exhibit a quite satisfactory relative activity of 66% even at 90°C.

This increased structural stability and expanded operating range exhibited by the immobilized enzyme at high temperatures is a direct result of the covalent immobilization of the enzyme onto the functionalized MNP surface via the glutaraldehyde cross‐linker (Behram et al. 2023). The multipoint covalent bonds between the free amino groups on the enzyme surface and the support material have imparted a high degree of physical rigidity to the enzyme's three‐dimensional tertiary structure (Kharazmi et al. 2020). This structural constraint significantly hinders the conformational unfolding and denaturation of the polypeptide chain that increased thermal energy could cause in the enzyme's active site (Kharazmi et al. 2020). This protective microenvironment formed by inorganic support materials around the enzyme acts as a shield, protecting the enzyme against thermal inactivation by raising the activation energy barrier that must be overcome for thermal denaturation (Navarro‐López et al. 2023; Rodrigues et al. 2021).

### 3.4 Optimum pH Assay

**FIGURE 6:**
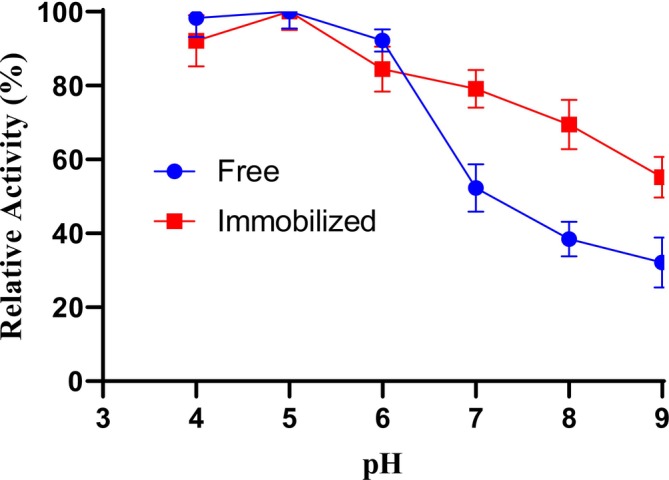
The effect of pH on the relative activity of free and immobilized pectinase (data are presented as mean ± SD of triplicates).

The effect of pH on the catalytic performance of free and immobilized pectinase were investigated in the range of 4.0–9.0. According to the experimental activity‐pH profile data presented in Figure 6, both free and immobilized pectinase reached maximum catalytic activity (100% relative activity) at pH 5.0, which is an acidic environment. This finding is consistent with studies reported in the literature showing that the optimal pH does not change after immobilization (Behram et al. 2023; Kharazmi et al. 2020). The fact that the immobilization process did not cause any shift in the enzyme's optimal pH value demonstrates that the microenvironment created by the modifying agents on the particle surface does not disrupt the ionization state of the critical amino acid residues in the enzyme's active site and maintains the protonation/deprotonation balance required for substrate binding at an ideal level (Dwevedi 2016; Zahirinejad et al. 2021).

When the optimum point is exceeded and environmental conditions are altered, structure‐based differences emerge in the responses of the two enzyme forms to pH changes. As the environment's alkalinity was gradually increased by moving away from the optimum pH value, a faster inactivation profile was observed in the relative activity of the free enzyme. While the relative activity of the free enzyme dropped to approximately 52% at pH 7.0, it fell to significantly lower levels 38% and 32%, respectively under highly alkaline conditions such as pH 8.0 and 9.0, thereby losing a significant portion of its catalytic function. In contrast, the developed magnetic nanobiocatalyst exhibited a markedly broader pH tolerance and superior operational stability compared to the free enzyme, particularly in neutral and alkaline regions (pH 7.0–9.0).

This expanded pH working range and increased alkaline resistance exhibited by the immobilized enzyme are a direct result of the enzyme molecules being anchored to the surface of inorganic nanoparticles functionalized via a glutaraldehyde cross‐linker through multipoint covalent bonds (Guisan et al. 2020; Zahirinejad et al. 2021). This strong chemical cross‐linking confers a high degree of conformational rigidity to the enzyme's three‐dimensional tertiary structure, thereby mechanically restricting the unfolding and molecular denaturation that could be caused by extreme H^+^ or OH^−^ ion concentrations in the environment (Guisan et al. 2020). Additionally, the buffering effect of the microenvironment created by the solid support material acts as a physical barrier that protects the enzyme by slowing the direct penetration of extreme pH fluctuations in the bulk solution into the enzyme's active site (Dwevedi 2016; Rodrigues et al. 2021).

### 3.5 Kinetic Parameters

To evaluate the catalytic efficiency of free and immobilized pectinase, reaction rates were measured at constant temperature and pH under varying pectin concentrations; the *K*_m_ and *V*_max_ parameters were calculated based on Michaelis–Menten kinetics and are presented in Table 1. According to the experimental results, the *K*_m_ value for free pectinase was determined to be 3.319 mg/mg and the *V*_max_ value was 0.280 U/mL. Following the immobilization process, these values were determined to be a *K*_m_ value of 3.940 mg/mL and a *V*_max_ value of 0.187 U/mL for the immobilized nanobiocatalyst.

**TABLE 1:** Kinetic parameters of free and immobilized enzyme (*K*_m_ and *V*_max_).

Enzyme	*K*_m_ (mg mL^−1^)	*V*_max_ (U/mL)
Free pectinase	3.319	0.280
Immobilized pectinase	3.940	0.187

The Michaelis constant (*K*_m_) is an inverse indicator of the enzyme's affinity for its substrate. Therefore, the increase in the *K*_m_ value observed during the transition from the free form to the immobilized form indicates a decrease in the enzyme's affinity for the pectin substrate (Behram et al. 2023; Miao et al. 2021). This situation may be due to the steric hindrances created around the enzyme's active site by inorganic solid support materials and glutaraldehyde, a cross‐linking agent (Miao et al. 2021). It may also stem from mass transfer (diffusion) limitations encountered by pectin a complex, high‐molecular‐weight polysaccharide as it moves from the bulk solution to reach the active site of the immobilized enzyme on the magnetic nanoparticle surface (Navarro‐López et al. 2023; Qi et al. 2020). Furthermore, the increase in *K*_m_ can also be explained by the structural rigidity gained by the enzyme as a result of multipoint covalent binding, which restricts the three‐dimensional conformational flexibility required for substrate binding and the induced‐fit mechanism (Miao et al. 2021; Qi et al. 2020).

On the other hand, the 33% decrease observed in the *V*_max_ value which represents the maximum reaction rate at full saturation (from 0.280 to 0.187 U/mL) is a direct result of the enzyme's covalent immobilization onto a solid surface (Muley et al. 2020). This decrease in the *V*_max_ value is attributed to a slowing of the catalytic turnover rate due to the high conformational rigidity imposed by the covalent bonds, and a shift in the reaction toward a diffusion‐controlled mechanism (Navarro‐López et al. 2023; Qi et al. 2020).

When evaluated from the perspective of industrial biocatalysis, these partial losses observed in catalytic parameters (an increase in *K*_m_ and a decrease in *V*_max_) as a result of covalent immobilization show strong parallels with similar studies in the literature (Behram et al. 2023; Miao et al. 2021; Muley et al. 2020). Furthermore, the primary purpose of enzyme immobilization is not to increase the enzyme's initial rate but to impart operational stability to the enzyme. The acceptable kinetic performance exhibited by the developed magnetic nanobiocatalyst, combined with its resistance to the extreme temperatures and pH fluctuations demonstrated in our study, its ease of recovery from the reaction medium using an external magnetic field (magnet), and its operational stability (reusability), translate into significant technological value. In large‐scale industrial processes requiring continuous flow, such as fruit juice processing, the extension of the enzyme's operational lifespan and the reduction in catalyst recovery costs more than compensate for this decrease in catalytic rate, demonstrating the system's economic feasibility.

### 3.6 Thermal Stability Assay

**FIGURE 7:**
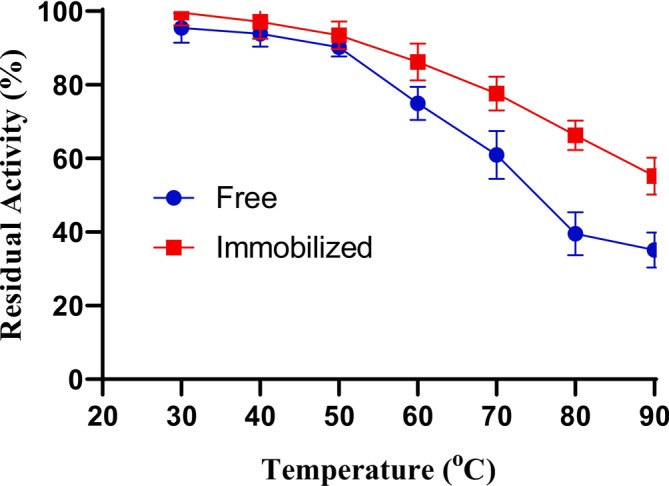
Comparative analysis of the thermal stability profiles of free and immobilized pectinase (data are presented as mean ± SD of triplicates).

One of the most critical parameters determining the operational feasibility and lifespan of industrial biocatalysts is the thermal stability of the enzyme (Rodrigues et al. 2021). The structural stability of free and immobilized pectinase was evaluated by incubating the enzymes in a pH 5.0 buffer at temperatures ranging from 30°C to 90°C for 3 h without the addition of substrate (Figure 7). Upon examination of the obtained thermal inactivation profiles, it was observed that at low temperature ranges (30°C–50°C), both enzyme forms were highly resistant to thermal denaturation and retained a large portion (over 90%) of their initial activity at the end of the third hour. However, when the incubation temperature was raised above 50°C, a progressive divergence in the thermal tolerances of the two enzymes was observed.

Free pectinase began to denature rapidly under increasing thermal stress; while it exhibited residual activity of approximately 75% and 60% at 60°C and 70°C, respectively, at 80°C the degradation of the enzyme's three‐dimensional structure accelerated, causing residual activity to drop to around 40%. At 90°C, the highest temperature tested, the free enzyme retained only 35% of its initial activity after a 3 h incubation. In contrast, the developed magnetic nanobiocatalyst demonstrated superior resistance to extreme thermal treatments compared to the free enzyme. The immobilized pectinase continued to exhibit high residual activity, maintaining approximately 86% at 60°C and 78% at 70°C. It demonstrated a thermally stable profile of industrial significance by retaining more than half (55%) of its residual activity even at extreme temperatures such as 80°C and 90°C.

This increased stability of the immobilized enzyme against prolonged exposure to high temperatures is a direct result of the enzyme being immobilized on the surface of an inorganic support material via a covalent cross‐linker (glutaraldehyde) (Barbosa et al. 2013). The multi‐point covalent bonds formed between the primary amine (‐NH_2_) groups on the enzyme surface and the functional groups of the support material have imparted a very high degree of conformational rigidity to the enzyme's three‐dimensional tertiary structure (Rodrigues et al. 2021). The unfolding and molecular denaturation process which, under normal conditions, is initiated by an increase in thermal energy breaking the non‐covalent weak interactions in the protein backbone has been prevented thanks to these strong physical and chemical constraints (Bai et al. 2023; Barbosa et al. 2013). This structural scaffold provided by the covalent bonds has slowed the kinetics of thermal inactivation by raising the thermal deactivation energy barrier that must be overcome for the enzyme to transition to its inactive form (Fang et al. 2016; Muley et al. 2020).

### 3.7 pH Stability Assay

**FIGURE 8:**
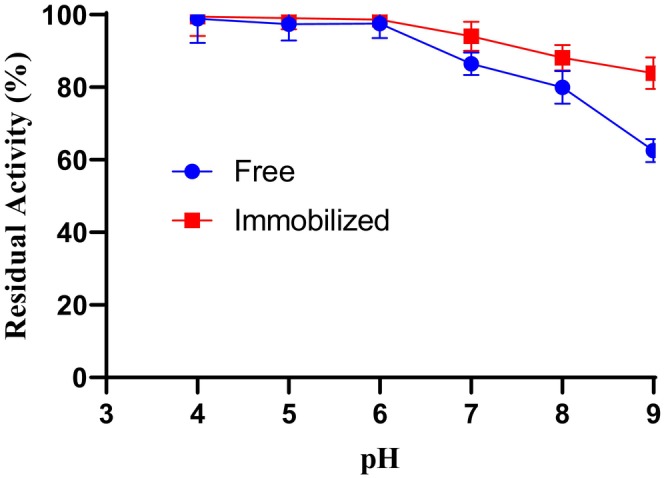
Comparative analysis of the pH stability profiles of free and immobilized pectinase (data are presented as mean ± SD of triplicates).

One of the most fundamental factors determining the operational lifespan and efficiency of industrial biocatalysts is their resistance to extreme conditions. The pH stability of free and immobilized pectinase was evaluated by incubating the enzymes in buffers with a pH range of 4.0–9.0 at 50°C for 3 h in a substrate‐free medium, followed by measurement of the remaining activity under optimal conditions. An examination of the inactivation profiles presented in Figure 8 reveals that both enzyme forms are highly stable under acidic and mildly acidic conditions (pH 4.0, 5.0, and 6.0) and retain a large portion (95%–100%) of their initial activity following 3 h of pH stress. However, as the medium pH shifted toward the neutral and alkaline ranges, a divergence emerged between the stabilities of the free and immobilized enzymes. A more rapid decline in the activity of the free pectinase was observed starting at pH 7.0. While the free enzyme retained approximately 86% of its initial activity at pH 7.0 and 80% at pH 8, it underwent severe inactivation at an alkaline extreme such as pH 9, exhibiting only 62% of its initial activity. In contrast, the developed magnetic nanobiocatalyst demonstrated better structural resistance and tolerance to alkaline stress conditions. The immobilized pectinase maintained a high level of residual activity, approximately 94% at pH 7.0 and 88% at pH 8, and managed to retain about 85% of its initial activity even at pH 9.

The rapid loss of activity of the free enzyme in neutral and alkaline environments is due to changes in the ionization state of amino acid residues in the enzyme structure caused by pH changes, and to the disruption of weak, non‐covalent interactions that stabilize the enzyme's tertiary structure (Minteer 2017). An increase in the concentration of OH^−^ ions in the medium led to irreversible conformational unfolding of the protein backbone and molecular denaturation. The remarkable pH stability exhibited by the immobilized enzyme, however, is a direct result of the multipoint covalent bonds formed between the support material and the residues on the enzyme's surface via the glutaraldehyde cross‐linker (Guisan et al. 2020). These strong chemical bonds impart a high degree of physical rigidity to the enzyme's three‐dimensional structure, thereby mechanically preventing the conformational disruptions triggered by changes in the environmental pH (Rodrigues et al. 2021). In addition, the enzyme's immobilization on the surface of the inorganic support material may have also served as a shield, slowing the direct penetration of extreme pH fluctuations in the bulk solution into the enzyme's microenvironment and protecting the enzyme like a solid buffer (Dwevedi 2016; Rodrigues et al. 2013).

### 3.8 Storage Stability Assay

**FIGURE 9:**
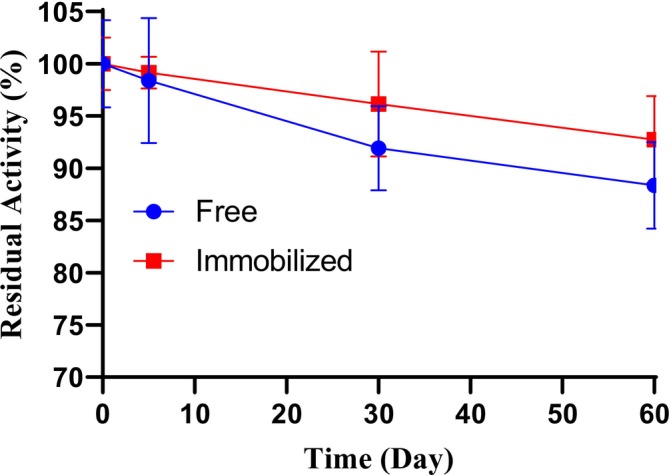
Comparative analysis of the storage stability profiles of free and immobilized pectinase (data are presented as mean ± SD of triplicates).

One of the most critical parameters determining the commercial and economic viability of industrial biocatalysts is their ability to maintain a high level of catalytic activity under long‐term storage conditions (shelf life) from production to the point of use (Homaei et al. 2013; Robescu and Bavaro 2025). Free enzymes tend to adopt catalytically inactive conformations in solution, undergo aggregation due to hydrophobic interactions, or degrade through autolysis (Barbosa et al. 2013; Rodrigues et al. 2021). The reason we chose pH 6.0 for storage stability is that both forms of the enzyme are highly stable at pH 6.0. Additionally, it provides a mild buffer environment that avoids extremely acidic or alkaline conditions which could denature the enzyme's conformational structure and minimizes structural stress caused by protonation and the enzyme's self‐degradation during prolonged incubation periods. To evaluate the operational lifespan of the developed magnetic nanobiocatalyst, the storage periods of both free and immobilized pectinase in a substrate‐free buffer solution were examined by measuring their activity at +4°C at the end of days 0, 5, 30, and 60, respectively. In the findings presented in Figure 9, the residual activity values were calculated as a percentage of the initial (day 0) activity.

Upon analysis of the data, it was found that both enzyme forms exhibited satisfactory stability at 4°C; however, the loss of activity over time for the immobilized pectinase was slower and more limited compared to that of the free enzyme. On the 5th day of storage, both the free and immobilized enzymes had retained nearly all (approximately 98% and 99%, respectively) of their initial activity. However, by the end of the 30th day, while the remaining activity of the free enzyme had declined to approximately 92%, the immobilized enzyme retained approximately 96% of its activity. At the end of the 60‐day extended storage period, the structural divergence between the two became much more pronounced. While the initial activity of the free pectinase dropped to ~88%, exhibiting a steady trend of inactivation, the magnetic nanobiocatalyst demonstrated better stability and managed to retain approximately 93% of its initial activity.

The superior structural stability exhibited by the immobilized enzyme during long‐term storage is a result of the enzyme molecules being anchored to the surface of functionalized magnetic nanoparticles via multipoint covalent bonds formed through glutaraldehyde (Guisan et al. 2020; Rodrigues et al. 2021). These strong chemical bonds formed between the solid support material and residues on the enzyme's surface (particularly the primary amine groups of lysine amino acids) have imparted a high degree of conformational rigidity to the enzyme's three‐dimensional tertiary structure (Guisan et al. 2020; Rodrigues et al. 2021). This rigidity has protected the enzyme from transitioning to an inactive form by mechanically restricting protein unfolding that could occur over time and due to solvent interactions (Zahirinejad et al. 2021). In addition, the inorganic support material's immobilization of the enzyme on a solid matrix prevents free enzyme molecules from moving freely in the solution phase and colliding with one another. Thus, it has successfully eliminated fundamental inactivation mechanisms—such as aggregation and intermolecular proteolysis (autolysis) commonly encountered in free proteins during storage (Rodrigues et al. 2021).

In the fruit juice industry, given the transfer and storage of biocatalysts as well as the waiting times between sequential operations, the ability of an enzyme to retain its activity for months without losing it represents a significant advantage in terms of process economics (Jothyswarupha et al. 2025).

### 3.9 Reusability Assay of the Immobilized Pectinase

In industrial‐scale biocatalytic processes, the high cost of enzymes and the difficulties in recovering them from the reaction medium are the primary factors limiting the economic viability of using free enzymes. The ability of immobilized enzymes to maintain their catalytic activity over successive reaction cycles that is, their operational stability (reusability) is one of the most important parameters determining the commercial and financial feasibility of the developed nanobiocatalyst (Nouri and Khodaiyan 2020; Taheri‐Kafrani et al. 2021). In this context, the reusability of the covalently immobilized pectinase was evaluated through hydrolysis cycles conducted using apple pectin under optimal conditions of pH 5.0 and 50°C.

**FIGURE 10:**
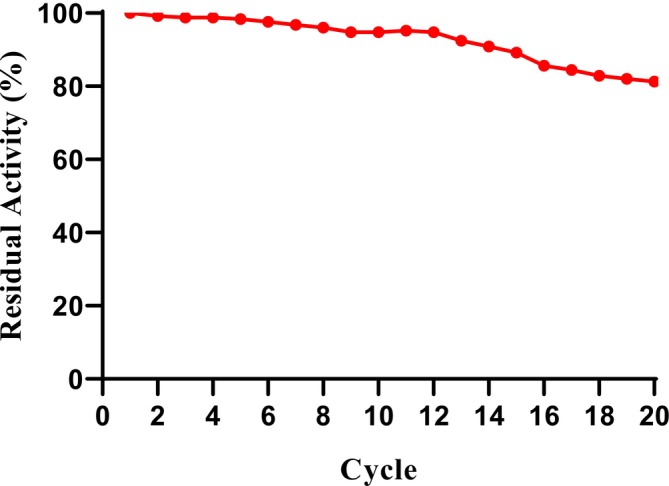
Reusability (operational stability) performance of immobilized pectinase.

An analysis of the operational stability data presented in Figure 10 reveals that the developed magnetic nanobiocatalyst exhibits exceptional performance throughout successive reaction cycles. The immobilized pectinase retained its initial activity almost entirely during the first few cycles and, in line with similar studies in the literature, managed to retain approximately 90% of its initial activity (residual activity) even at the end of the 15th usage cycle. At the end of 20 cycles, it exhibited 81% residual activity.

The fundamental mechanism underlying this high level of residual activity exhibited by the immobilized enzyme during successive cycles is the strong covalent bonds formed between the functionalized magnetic nanoparticles and the pectinase via glutaraldehyde. This multipoint covalent bonding physically and chemically prevents the enzyme from leaching from the particle surface into the solution during the washing and recovery stages (Fang et al. 2016; Mosafa et al. 2014). Furthermore, instead of methods that cause intense mechanical stress such as traditional filtration or centrifugation, the ability to easily, quickly, and losslessly separate the nanobiocatalyst from the reaction medium within a short time using an externally applied magnetic field (magnet) has minimized physical attrition of the support material and the resulting enzyme losses (Fang et al. 2016; Mosafa et al. 2014).

The approximately 10% loss of activity observed as cycles progress is an expected and acceptable phenomenon for industrial biocatalysts. This partial inactivation results from cumulative mechanical damage caused by continuous magnetic stirring and washing during successive cycles, the accumulation of substrate macromolecules or hydrolysis products in the pores or on the surface of the particles over time, creating steric hindrance to active sites, and minor conformational distortions resulting from the enzyme's prolonged exposure to operating conditions (Fang et al. 2016; Kharazmi et al. 2020; Miao et al. 2021). In addition, the removal of very small amounts of physically adsorbed enzyme from the medium during the washing steps may also have contributed to this decline (Kharazmi et al. 2020).

These findings regarding operational stability demonstrate that the synthesized functional magnetic nanobiocatalyst not only enhances the enzyme's structural stability but also significantly reduces enzyme consumption, thereby offering an extremely economical, sustainable, and high‐performance alternative for continuous or batch reactor systems used in fruit juice clarification processes.

### 3.10 Clarification of Grape Juice

Freshly squeezed fruit juices exhibit a highly turbid and viscous texture due to the presence of colloidal polysaccharides, such as pectin, which are released into the liquid phase as a result of the breakdown of cell walls and the middle lamella (Nouri and Khodaiyan 2020; Taheri‐Kafrani et al. 2021). In a naturally acidic environment, these negatively charged pectin substances wrap around protein particles in the fruit juice, creating electrostatic repulsive forces between the particles and thus forming a stable haze layer that prevents sedimentation in the fruit juice (Kuddus 2019). To eliminate this haze, which directly affects consumer acceptance and commercial value, the performance of a covalently immobilized magnetic pectinase nanobiocatalyst on grape juice was investigated over a 3‐h (180‐min) period at 50°C.

**FIGURE 11:**
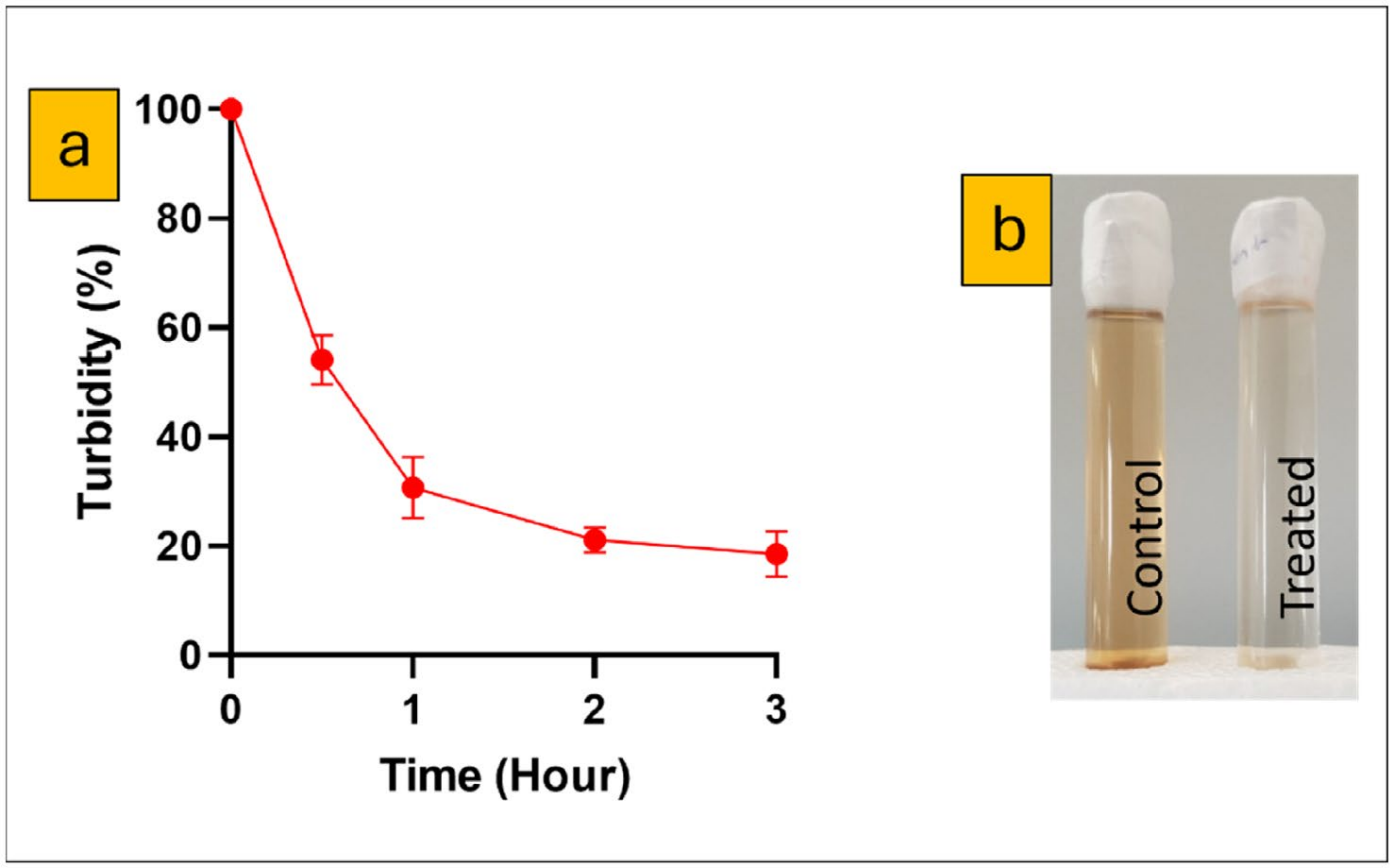
Quantitative and macroscopic analysis of the performance of immobilized pectinase in the grape juice clarification process. (a) Time‐dependent relative turbidity profile of grape juice treated with the immobilized enzyme during a 3‐h enzymatic hydrolysis at 50°C (data are presented as mean ± SD of triplicates). (b) Visual comparison of grape juice samples from the control group (Control), which was subjected to the same conditions without any enzyme addition, and the group (Treated) in which the juice was hydrolyzed using a magnetic nanobiocatalyst, at the end of the 3‐h incubation period.

According to the turbidity graph presented in Figure 11 (Figure 11a), there was a dramatic decrease in the turbidity of the grape juice treated with the nanobiocatalyst over time. During the first 30 min, when enzymatic hydrolysis was at its fastest, the turbidity value dropped to 55% of the initial level. By the end of the first hour (60 min), this value had fallen to approximately 30%, demonstrating a significant rate of clarification. As the reaction progressed, the enzymatic reaction rate gradually slowed due to the decreasing amount of hydrolyzable substrate in the medium, and by the end of the third hour, the turbidity of the grape juice had decreased by more than 80% compared to the initial value, reaching 18%.

These quantitative data, measured spectrophotometrically at 660 nm, are clearly corroborated by the macroscopic images presented in Figure 11b. While the control sample, which was not subjected to any enzymatic treatment and was incubated under exactly the same thermal conditions (50°C, 3 h), retained its dark‐colored, dense, and turbid colloidal structure, the sample treated with immobilized pectinase (Treated) acquired a highly transparent, clear, and sediment‐free appearance.

This high clarification capacity observed is a direct result of the immobilized pectinase specifically hydrolyzing the α‐(1,4)‐glycosidic bonds in the main backbone of the pectin molecules suspended in grape juice (Cosimo et al. 2013; Kuddus 2019). When pectin polymers were broken down into smaller, soluble oligomers and galacturonic acid monomers, the protective pectin shell that stabilized the haze was disrupted, exposing positively charged proteins within. With the elimination of the electrostatic repulsive forces between oppositely charged particles, cloud particles coalesced through flocculation to form precipitates, resulting in the clarification of the liquid phase (Kuddus 2019).

From an industrial perspective, in traditional free‐enzyme applications in the liquid phase, thermal inactivation processes are carried out at high temperatures because the enzyme cannot be separated from the product after the reaction. This leads to the degradation of the fruit juice's natural color, vitamins, and delicate aroma components (Patel et al. 2022). The functionalized magnetic nanobiocatalyst synthesized in our study not only effectively bleaches grape juice with high catalytic efficiency but can also be easily removed from the liquid within seconds at the end of the reaction using an externally applied magnetic field (magnet) (Bilal, Zhao, et al. 2018). This feature not only protects the nutritional value and quality of the fruit juice from thermal degradation but also enables the enzyme to be recovered and reused multiple times, thereby offering an economical and sustainable technological solution for large‐scale, continuous production facilities (Jothyswarupha et al. 2025; Vaghari et al. 2016).

## 4 Conclusions

One of the obstacles to the use of enzymes such as pectinases has been the high costs associated with their production and purification. This study has shown that immobilizing pectinase on functionalized magnetic nanoparticles (MNPs) can help reduce the high cost associated with free enzyme by enabling the reuse of biocatalysts through rapid magnetic separation. The immobilized enzymes exhibited good activity and were recoverable after 20 consecutive hydrolysis cycles. Furthermore, the immobilized enzymes showed better tolerance to pH and temperature changes compared to free enzymes. The storage stability of the immobilized enzyme was better than that of the free enzyme. It is important to note that it exhibited a high reusability rate of 81% after 20 consecutive uses in pectin hydrolysis. This can be considered to have significant potential for reducing costs and increasing the usability of immobilized pectinase for industrial applications.

## Author Contributions

**Olgun Cirak:** methodology, investigation, visualization, writing – original draft, writing – review and editing, resources, validation. **Mesut Işık:** investigation, writing – review and editing, data curation, methodology, resources. **Alev Akpinar Borazan:** resources, investigation, writing – review and editing. **Şükrü Beydemir:** project administration, writing – review and editing, supervision.

## Conflicts of Interest

The authors declare no conflicts of interest.

## Data Availability

Data will be made available on request.
